# Prevalence of risk factors for diabetic foot complications

**DOI:** 10.1186/1471-2296-8-59

**Published:** 2007-10-10

**Authors:** Fatma Al-Maskari, Mohammed El-Sadig

**Affiliations:** 1 Department of Community Medicine, Faculty of Medicine & Health Sciences, United Arab Emirates University, Al-Ain, PO Box 17666, U.A.E

## Abstract

**Background:**

Foot complications are common in diabetic patients and are considered one of the most expensive diabetes (DM) complications to treat. The aim of this study was to determine the prevalence and risk factors for foot complications among diabetic patients in Al-Ain district, United Arab Emirates (UAE).

**Methods:**

The study was part of a general cross-sectional survey carried out to assess the prevalence of DM complications in Al-Ain district, UAE. A sample of 513 diabetic patients with a mean age of 53 years (SD: ± 13) were randomly selected during 2003/2004. All completed an interviewer-administered questionnaire and underwent medical assessment including foot examination and assessment of presence of peripheral neuropathy (PN) and peripheral vascular disease (PVD).

**Results:**

Forty nine percent of the study populations were diagnosed to have DM without presenting with symptoms of diabetes and 35% had hypertension. The majority (86%) had type 2 DM. Of the total sample, 39% (95% CI: 35.1-43.7%) had PN and 12% (95% CI: 8.8–14.4%) had PVD. There were no cases of amputation and only one case had previous history of lower extremity ulceration. Significant risk factors for PN and PVD were: male gender, poor level of education, UAE nationality, increased duration of diabetes, type 2 DM, presence of hypertension and microalbuminuria (MA).

**Conclusion:**

Despite the low prevalence of foot ulceration and amputation among the study population, nevertheless, a substantial proportion had potential risk factors for foot complications.

## Background

Peripheral neuropathy (PN) and peripheral vascular disease (PVD) are well known common long-term complications of diabetes, and although a proportion of people with PN and PVD have severe and debilitating pain, many are asymptomatic [1]. However, despite the lack of symptoms, people with PN and PVD are known to be at high risk of foot complications including foot ulceration, infection and amputation [2-4]. PN and PVD are the main causes of non-traumatic lower limb amputation [5-7].

Complications affecting the lower limbs are among the most common manifestations of diabetes; it was reported that 15% of diabetic patients will eventually suffer from foot ulceration during their lifetime [8]. These complications are a frequent cause of hospitalization and disability; with 1 in 5 hospitalizations among diabetics directly related to foot ulcers [9]. Moreover, according to some conservative estimates, the treatment costs of these complications account for approximately 25% of total hospital costs of diabetes care, the true costs of which might be an order of magnitude higher [10].

It is generally upheld that effective prevention of diabetic foot requires in-depth knowledge of the pathogenesis and clinical correlates of the complication; neither of which is well understood so far [11]. Targeting patients at increased risk for developing foot ulcer is believed to constitute a cost effective strategy to control progression to end stage complications. Evidence in the literature suggests that the early detection and treatment of diabetic foot complications could reduce the prevalence of ulceration by 44% to 85% [12,13].

The International Diabetes Federation (IDF) in 2003 ranked the UAE's prevalence rates of type 2 DM and impaired glucose tolerance (IGT) as the second highest in the world (20% for DM and 26% for IGT) [14]; a serious status which indicates the extent and gravity of the problem in the UAE population. Indeed, the disease and its complications such as PVD and PN are likely to constitute a sizable burden to the UAE society, the magnitude of which is not yet known. To our best knowledge, this is the first epidemiological study assessing the prevalence and risk factors of foot complications among diabetic patients in the UAE population. As such, it is hoped that the study would lay the grounds for future efforts aiming to control the problem.

## Methods

### Overall Design

The study was part of a general cross-sectional survey of DM patients carried out to assess and establish the prevalence of DM complications including foot complications among diabetic patients in the Al-Ain District, UAE. The district is located in the interior lands of Abu Dhabi Emirate and constitutes the second largest district there; with an approximate population of 500,000 according to recent estimates in the UAE.

### Subjects and Setting

The sampling frame of this study included all UAE and non-UAE diabetic patients of all ages and both genders, attending any PHCC for any reason or any diabetic clinic at hospitals for follow up in Al-Ain district. Sample size estimations were based on the general requirement that prevalence estimates should have an (absolute) standard error of no more than 0.02 (2%). Accordingly, a sample size of 625 was calculated. In order to obtain a representative sample, multistage random sampling was used. To achieve that all primary health care centers (PHCC) and diabetic clinics in the district were listed and enumerated and from these 8 PHCC were selected (out of 22 rural and urban PHCC), in addition to two diabetic clinics at the two main public hospitals of Al-Ain. In the absence of a diabetes registry or a computerized database for patients in the district, systematic random sampling within these units (centers/clinics) was used to select patients to be approached for participation in the study. Thus, every third DM patient visiting any of the participating centers and clinics was approached. In total 600 patients were approached by general practitioners and diabetologists, out of whom 513 (86%) agreed to enroll. The study was approved by the Ethics Committee of the Faculty of Medicine and Health Sciences of the UAE University.

### Data Collection

After receiving informed consent, diabetic patients were interviewed by their treating doctors about DM type, duration, treatment profile, level of control, presence or absence of chronic DM complications including previous ulcer/amputation of the lower limbs and presence of vascular symptoms of cramps and/or claudications, neuropathic symptoms of tingling, numbness, and burning sensation with a 'stocking and glove' distribution. Additionally, the research nurse took measurement of patients' blood pressure early in the morning and prior to drawing blood samples while in the sitting position, using a standard mercury sphygmomanometer. The WHO definition of hypertension was used in this study: systolic blood pressure 160 mmHg or more and/or a diastolic blood pressure 95 mmHg or more, or on going treatment with antihypertensive drugs. Height was measured without shoes, and weight recorded while wearing indoor clothing. Body mass index (BMI) (weight in Kg, divided by the square of the height in m^2^) was calculated. The WHO (1977, 1979) classification for BMI was used to estimate the degree of obesity [15]. A standard 12- lead electrocardiogram (ECG) was recorded for all patients. Fasting blood samples were taken to assess lipid profile, blood sugar and glycated hemoglobin (HbA_1C_) levels. Total lipid profile (total cholesterol (TC), high density lipoprotein (HDL), TC/HDL Ratio, low density lipoprotein (LDL) and triglycerides) was measured by a capillary tube whole blood method using the Cholesterol LDX lipid analyzer. Dyslipidaemia was taken to be present when the total cholesterol was >5.60 mmol/L and/or triglycerides >2.10 mmol/L, and/or LDL >3.4 mmol/L, and or HDL <0.91 mmol/L. Glycated haemoglobin (HbA_1C_) was measured using the Bayer DCA 2000+ analyzer and a value of less than 7% was taken to indicate good glycemic control. Urinary albumin concentration was measured by using semi-quantitative dry immuno chemical screening strips (Micral 11^® ^test strips (Roche diagnostic GmbH Mannheim Germany). Micral Tests were performed on first morning urine collections and a value of more than 20 μg/min was judged as pathological.

Both feet were examined for signs of vasculopathy and neuropathy including skin status (color, thickness, dryness, cracking, trophic changes, decreased hair growth), the nail status (color, thickness, trophic changes), and the presence of deformities (such as claws and hammer toes), oedema, infection, ulceration, callus and blistering. Gangrene and amputation were also noted.

PN was assessed by vibratory, monofilament, muscle strength and tendon reflex testing. Pressure, pain, vibration and joint position sensitivities were evaluated bilaterally. For pressure perception, the 10 g Semmes-Weinstein monofilaments was used on 4 sites of the foot. These sites were without callus, notably the pulps of the hallux and metatarsal heads of first, third and fifth toes. The site was considered sensate if the patient responded, "yes" upon contact with the monofilament and insensate if there was no response.

For vibration perception, a 128 Hz tuning fork was applied at 3 sites on the foot, the pulp of the hallux, the lateral and the medial malleoli. The patient was asked to describe what he felt. If he/she described a feeling of vibrations, the site concerned was considered normal. If he/she described anything other than vibrations, the site concerned was considered abnormal. In addition, pin-prick perception (using standard neurotips) on the dorsal surface of the great toe and the index finger were evaluated. Neuropathy was further assessed by examining the tendon reflexes bilaterally and testing for muscle strength by examining for extension of the knee and dorsiflexion of the foot.

Diabetic Neuropathy Symptoms (DNS) [16] along with the Diabetic Neuropathy Examination (DNE) scores [17] were used together to define and assess neuropathy. Neuropathy was considered to be present if DNS score was >0 and/or the DNE score was >3. Lower limb ischemia was ascertained by the examining physician through palpation of the dorsalis pedis and the tibialis posterior pulses when one or more foot pulses were judged absent with or without symptoms of lower-limb claudication and/or amputation or gangrene were present.

### Statistical Analysis

The data was coded and processed on IBM compatible computers, using the Statistical Package for Social Sciences (SPSS) software (version 14). Descriptive analysis, using standard statistical methods was performed. Chi-square tests or Fisher's exact test, independent t-tests and Pearson correlation coefficient were used to ascertain the association between the outcome variables of foot complications and their determinants; specifically relevant clinical variables.

A multiple logistic regression model with backward selection (criterion: p-value to remove ≥ 0.10) was used to estimate the simultaneous effect of several determinants of neuropathy (PN) and peripheral vascular disease (PVD) among the sample population. This method first enters all independent variables selected by the researcher in the logistic equation and then sequentially removes them, starting with the least significant one, until all p-values are below the chosen value 0.10 (p-value to remove).

For PN and PVD, the variables included as predictors were sex, level of education (primary, intermediate, secondary and university), age groups (categorical intervals in years), type of DM, duration of disease (categorical intervals in years), nationality groups (UAE, Gulf citizens, Asians, Arabs of other nationalities and others). That is in addition to a number of determinants of dichotomous (yes/no) outcome of PN including presence of retinopathy, MA and glycemic control and of PVD including presence of hypertension, MA and glycemic control. Missing values (very few) for variables were imputed using mean imputation, i.e. they were substituted by the variable mean.

Analysis of variance (ANOVA), was used to estimate overall model performance, while estimated model standard errors and *t*-tests were used to test for the significance of individual (linear) regression parameters.

## Results

### Socio-Demographic Characteristics of the Study Population

The descriptive analysis of the sample showed that 52% were males, 27% were aged 60 years or above, 75% were UAE nationals and most of patients (63%) were illiterate. Of the total sample 12.8% (95% CI: 11.0–14.6) were current smokers while 8.2% (95%CI: 6.7–9.7) were ex-smokers (Table 1).

**Table 1:** Socio-Demographic Characteristics of DM Patients in Al-Ain District, UAE, 2003–2004 (n = 513)

**Variable name**	**Prevalence of DM**	
	**n**	**Percent (95% CI)**

**Sex**		
Male	264	51.5 (47.2–55.8)
Female	249	48.5 (44.2–52.8)
**Level of Education**		
Illiterate	318	62.8 (58.6–67.0)
Completed primary school	99	19.6 (16.1–23.1)
Completed secondary school	60	11.9 (9.1–14.7)
Completed university	29	5.7 (3.7–7.7)
**Age group (Years)**		
40 or less	81	15.8 (12.6–19.0)
41 – 49	137	26.8 (23–30.6)
50 – 59	154	30.1 (26.1–34.1)
60 or above	140	27.3 (23.4–31.2)
**Nationality group**		
UAE	382	74.6 (70.8–78.4)
Arabs from Gulf countries	54	10.5 (7.8–13.2)
Arabs from other countries	54	10.5 (7.8–13.2)
Asians	22	4.3 (2.5–6.1)
**Smoking**		
Current smoker	64	12.8 (11.0–14.6)
Ex smoker	41	8.2 (6.7–9.7)
**BMI Group**		
Under weight (<18.5)	6	1.2 (0.2–2.2)
Healthy weight (18.5–24.99)	113	22.5 (18.8–26.2)
Overweight (25–29.99)	195	38.8 (34.5–43.1)
Obese (>30)	188	37.7(33.3–41.7)

### Clinical Characteristics

Of the total sample, the majority (86%) had Type 2 DM, 49% were diagnosed incidentally and most of them (79%) had the disease for ≥ 10 years. Of the total population 35% (95%CI: 30.8–39) had hypertension, 76% were obese or overweight and 61% (95% CI: 56.7–65.7) had microalbuminuria (MA) (Table 2). The majority of patients (84%) partially managed the disease by oral hypoglycemic agents, 24% by insulin while two thirds were not following any exercise regime as part of their routine management. The analysis of glycemic control using HbA_1C _showed that 38% (95%CI: 32.8–42.4) had good control. High total cholesterol was present in 34.4% (95%CI: 30.0–38.8), high triglycerides in 25.2% (95%CI: 21.1–29.3), high LDL in 53.4% (95%CI: 44.9–61.9) while low HDL was present in 25.7% (95%CI: 18.5–32.9) of the study population (Table 2).

**Table 2:** Clinical Characteristics of DM Patients in Al-Ain District, UAE, 2003–2004 (n = 513)

	**n**	**Percent (95% CI)**
**Type of DM**		
Type 1	68	13.6 (10.6–16.6)
Type 2	431	86.4 (83.4–89.4)
**Mode of Diagnosis**		
Incidental	245	48.5 (44.1–52.9)
Screening	39	7.7 (5.4–10.0)
Symptomatic	221	43.8 (39.5–48.1)
**Family History of DM**		
Present	270	54.3 (49.9–58.7)
Absent	227	45.7(41.3–50.1)
**Duration of the Disease**		
< 1 year	33	6.6 (4.4–8.8)
1–5 years	199	40.0 (35.7–44.3)
6–10 years	161	32.3 (28.2–36.4)
11–20 years	98	19.7 (16.2–23.4)
>21 years	7	1.4 (0.4–2.4)
**Hypertension**		
Present	178	34.9 (30.0–38.8)
Absent	332	65.1(61.0–69.2)
**Total Cholesterol**		
High (>5.60 mmol/L)	152	34.4 (30.0–38.8)
Normal (≤ 5.60 mmol/L)	290	65.5(61.1–69.9)
**Triglycerides**		
High (>2.10 mmol/L)	105	23.9 (19.9–27.9)
Normal (≤ 2.10 mmol/L)	334	76.1(72.1–80.1)
**HDL**		
High (≥ 0.91 mmol/L)	104	74.3 (67.1–81.5)
Low (<0.91 mmol/L)	36	25.7 (18.5–32.9)
**LDL**		
High (>3.4 mmol/L)	70	53.4(44.9–61.9)
Normal (≤ 3.4 mmol/L)	61	46.6(38.1–55.1)
**Microalbuminuria**		
Present (>20 μg/min)	276	61.2 (56.7–65.7)
Absent (<20 μg/min)	175	38.8 (34.3–43.3)
**HBA**_**1**_**c**		
Good control (<7%)	150	37.6(32.8–42.4)
Poor control (>7%)	246	62.4(57.6–67.2)
**Peripheral Neuropathy**		
Present	199	39(35.1–43.7)
Absent	306	61(56.3–64.9)
**Peripheral Vascular Disease**		
Present	59	12(8.8–14.4)
Absent	451	88(85.6–91.2)

### Prevalence of Foot Complications

Of the total sample, 39% (95% CI: 35.1–43.7%) had PN and 12% (95% CI: 8.8–14.4%) had PVD (Table 2). Trophic skin and nail abnormality were present in 3% (95% CI: 1.4–4.4%) of the sample population and symptoms including cramp-like-pain in legs or feet, tingling, numbness, and burning sensations with a "stocking and glove distribution" were frequently reported in 35% (95% CI: 30.4–38.6%) of them. There were no cases of foot deformity or amputation and only one case had previous history of lower extremity ulceration.

The risk factors for neuropathy, predicted using univariate analysis were: male gender (p = 0.006), age (p = 0.01), disease duration (p = 0.0001), poor level of education (p = 0.04), poor glycemic control (p = 0.006) and type 2 DM (p < 0.006). The complication was also highly significantly associated with other microangiopathic complications such as diabetic retinopathy (p = 0.001) and presence of microalbuminuria (p = 0.01) (Table 3).

**Table 3:** Peripheral Neuropathy (PN) in relation to Baselines Characteristics of the Study Population in Al-Ain District, UAE, 2003–2004 (n = 513)

**Variable name**	**Presence of Neuropathy**		**Absence of Neuropathy**		
	**n**	**Percent (95% CI)**	**n**	**Percent (95% CI)**	**p-value**

**Sex**					0.006
Male	118	59.3(52.47–66.13)	143	46.7(41.1152.29)	
Female	81	40.7(33.87–47.53)	163	53.3(47.7158.89)	
**Level of Education**					0.040
Illiterate	116	59.5(52.6–66.4)	197	65(59.6–70.4)	
Completed primary school	33	16.9(11.6–22.2)	64	21.1(16.5–25.7)	
Completed secondary school	32	16.4(11.2–21.6)	27	8.9(5.7–12.1)	
Completed university	14	7.2(3.6–10.8)	15	5(2.5–7.5)	
**Age group (Years)**					0.015
40 or less	23	11.6(7.2–16.0)	57	18.7(14.3–23.1)	
41 – 49	46	23.1(17.2–29.0)	91	29.8(24.7–34.9)	
50 – 59	72	36.2(29.5–42.9)	80	26.2(21.3–31.1)	
60 or above	58	29.1(22.8–35.4)	77	25.2(20.3–30.1)	
**Nationality group**					0.014
UAE	140	70.7(64.36–77.04)	237	77.5(72.82–82.18)	
Arabs from Gulf countries	16	8.1(4.30–11.90)	35	11.4(7.84–14.96)	
Arabs from other countries	31	15.7(10.63–20.77)	23	7.5 (4.55–10.45)	
Asians	11	5.6(2.40–8.80)	11	3.6 (1.51–5.69)	
**Type of DM**					0.006
Type 1	37	19.2	31	10.4(6.93–13.87)	
Type 2	156	80.8	267	89.6(86.13–93.07)	
**Duration of the Disease**					0.000
< 1 year	15	7.6	17	5.8(3.12–8.48)	
1–5 years	54	27.3	143	49(43.27–54.73)	
6–10 years	68	34.3	90	30.8(25.50–36.10)	
11–20 years	57	28.8	39	13.4(9.49–17.31)	
>21 years	4	2	3	1(0.00–2.14)	
**Retinopathy**					0.000
Present	35	30.4	31	13.8(9.29–18.31)	
Absent	80	69.6	194	86.2(81.69–90.71)	
**Microalbuminuria**					0.012
Present (>20 μg/min)	118	69.4	157	42.5(36.64–48.36)	
Absent (< 20 μg/min)	52	30.6	116	57.5(51.64–63.36)	
**HAB**_**1C**_					0.006
Good control (<7%)	50	33.1	144	40.7(34.52–46.88)	
Poor control (>7%)	101	66.9	99	59.3(53.12–65.48)	

Predicted risk factors for PVD, using univariate analysis were: male gender (p < 0.001), age (p < 0.001), incidental diagnosis of diabetes (p = 0.006), duration of diabetes (p < 0.001) and presence of hypertension (p = 0.01). The complication was also highly significantly associated with other chronic complications of diabetes such as retinopathy (p = 0.009), cerebrovascular disease (p = 0.001) and presence of coronary artery disease (p = 0.01) (Table 4).

**Table 4:** Peripheral Vascular Disease (PVD) in Relation to Baseline Characteristics ofthe Study Population in Al-Ain District, UAE, 2003–2004 (n = 513)

**Variable name**	**Presence of PVD**		**Absence of PVD**		
	**n**	**Percent (95% CI)**	**n**	**Percent (95% CI)**	**p-value**

**Sex**					
Male	44	74.6(63.4985.71)	217	48.1(43.4952.71)	0.000
Female	15	25.4(14.2936.51)	234	51.9(47.2956.51)	
**Age group (Years)**					
40 or less	3	5.1(0.00–10.71)	78	17.3(13.81–20.79)	0.000
41 – 49	8	13.6(4.85–22.35)	129	28.7(24.52–32.88)	
50 – 59	16	27.1(15.76–38.44)	136	30.2(25.96–34.44)	
60 or above	32	54.2(41.49–66.91)	107	23.8(19.87–27.73)	
**Duration of the Disease**					
< 1 year	1	1.7(0.00–5.00)	31	7.1(4.69–9.51)	0.000
1–5 years	12	20.3(10.04–30.56)	186	42.7(38.06–47.34)	
6–10 years	2	35.6(23.38–47.82)	140	32.1(27.72–36.48)	
11–20 years	23	39(26.55–51.45)	74	17(13.47–20.53)	
>21 years	2	3.4(0.00–8.02)	5	1.1(0.12–2.08)	
**Mode of diagnosis**					
Incidental	38	67.9(55.67–80.13)	206	46.2(41.57–50.83)	0.006
Screening	1	1.8(0.00–5.28)	38	8.5(5.91–11.09)	
Symptomatic	17	30.4(18.35–42.45)	203	45.3(40.68–49.92)	
**Retinopathy**					
Present	16	33.3(19.97–46.63)	51	17.2(12.91–21.49)	0.009
Absent	32	66.7(53.37–80.03)	246	82.8(78.51–87.09)	
**Coronary Artery Disease**					
Present	17	28.8(17.25–40.35)	56	12.6(9.52–15.68)	0.001
Absent	42	71.2(59.65–82.75)	389	87.4(84.32–90.48)	
**Cerebrovascular disease**					
Present	5	8.6(1.38–15.82)	12	2.7(1.20–4.20)	0.018
Absent	53	91.4(84.18–98.62)	435	97.3(95.80–98.80)	
**Hypertension**					
Present	29	49.2(36.44–61.96)	148	33(28.65–37.35)	0.015
Absent	30	50.8(38.04–63.56)	300	67(62.65–71.35)	

The multivariate stepwise logistic regression analysis with backward selection of PN on variables including sex, level of education, age group, nationality group, type of DM, duration of disease, presence of retinopathy, MA, and HbA_1c_, showed that PN was only significantly associated with increased duration of disease (p = 0.001), UAE nationality (p = 0.02) and presence of MA (p = 0.01) (Table 5). It is worthy noting that MA remained significantly associated with PN (p = 0.01) after adjusting for age, type of DM, HbA_1c_, level of education and nationality (logistic regression adjusted OR: 0.521; 95% CI: 0.313–0.866). However, the MA effect disappeared when adjusted for duration of disease, age group, type of DM, HbA_1c_, level of education and nationality. The effect of increased duration of disease remained highly significantly associated with neuropathy (p = 0.002) even after adjusting for nationality, presence of MA, HbA_1_c, education, sex, age and nationality (logistic regression adjusted OR: 0.670; 95% CI: 0.520–0.864).

**Table 5:** Multivariate Analysis of Risk Factors for PN among DM Patients in Al-Ain District, UAE, 2003–2004 using stepwise Logistic Regression

**Variable**	**Regression coefficient**	**P value**	**Adjusted OR**	**95% CI**
UAE Nationality	-0.299	0.029	0.741	(0.567–0.970)
Increased DM duration	-0.431	0.001	0.650	(0.502–0.842)
Presence of MA	-0.651	0.013	0.522	(0.312–0.871)

The multivariate stepwise logistic regression analysis with backward selection of PVD on variables including sex, age group, level of education, duration of disease, mode of diagnosis, type of DM, presence of hypertension, HbA_1c _and presence of MA revealed that only male, (adjusted OR: 0.275; 95% CI: 0.124 – 0.611), increased duration of disease (adjusted OR: 0.441; 95% CI: 0.288 – 0.676), low level of education (adjusted OR: 2.869; 95% CI: 1.448 – 5.685), type 2 DM (adjusted OR: 0.110; 95% CI: 0.014 – 0.882), presence of hypertension (adjusted OR: 2.361) were significantly associated with PVD (Table 6).

**Table 6:** Multivariate Analysis of Risk Factors for PVD among DM Patients in Al-Ain District, UAE, 2003–2004 using stepwise Logistic Regression

**Variable**	**Regression coefficient**	**P value**	**Adjusted OR**	**95% CI**
Male gender	-1.289	0.002	0.275	(0.124–0.611)
Increased DM duration	-0.818	0.000	0.441	(0.288–0.676)
Low Level of education	1.054	0.003	2.869	(1.448–5.685)
Type 2 DM	-2.208	0.038	0.110	(0.014–0.882)
Presence of hypertension	0.859	0.023	2.361	(1.125–4.955)

## Discussion

The present study is the first study in the UAE investigating the risk factors of diabetic foot complications. As proved elsewhere, our results, clearly demonstrate that DM eventually lead to chronic complications, including PVD and PN. It is known that PVD and PN are potential risk factors for foot complications. Indeed, with the high prevalence rates of DM in the UAE population and the high rates of PVD and PN among patients which has been revealed in this study, it is vital to observe for diabetes foot complications.

The results of this study showed that the overall prevalence of PN was 39%, which was higher than the equivalent rates reported in other populations [7,18-21]. Comparatively, the rate revealed for PVD (12%) in the UAE population was far lower than that reported in other populations [22-24]. However, the fact that 35% (95% CI: 30.4–38.6%) of the sample population reported to have symptoms of neuropathy and/or vasculopathy and 3% had trophic skin and nails, is a clear indication that they are at increasing risk of developing foot complications in the future. The high prevalence of PN compared with the relatively low prevalence of PVD in the study population can be due to methodological biases for diagnosing neuropathy. Although the sensitivity and specifity of the DNS score were high when defined using other standard clinical methods [16]. However, symptom scores may be less reliable due to their subjectivity.

The results also showed that history of previous leg ulceration, deformity, gangrene and amputation were infrequently reported in the population; an issue which might reflect a systematic sampling bias due to the fact that the majority of the sample populations (82%) were recruited from primary health care centers (PHCC). It is known and naturally expected that diabetic patients with severe complications tend to report and follow up in hospitals and not in PHCC.

The multivariate logistic regression analysis further showed that the main risk factors for PN and PVD and thus potentially for foot complications were male gender, poor level of education, UAE nationality, increased disease duration (10–12 years), type 2 DM, presence of hypertension and MA. The results are consistent with findings elsewhere [18,25-29]. It is known that the risk of ulcers and lower limb amputations is higher in males, in patients with diabetes duration of 10 years or more, those who have poor glycemic control or have other cardiovascular, retinal or renal complications [30]. Indeed, the fact that the majority of the surveyed population (63%) were illiterate and that 62% of them were of poor glycemic control; and, the fact that poor foot care is common among patients and practitioners as well, as reported elsewhere [31-33], reveal the extent, gravity and complexity of the problem in the UAE.

It is interesting to note that MA was the only modifiable risk factor for PN, revealed in the study and the result is consistent with similar studies elsewhere [20,22,34]. However, some studies suggest that increased urinary albumin might be a marker for endothelial damage, the function of which may be the common cause of both microalbuminuria and diabetic neuropathy [35,36].

Similarly, the analysis revealed that hypertension is the only modifiable risk factor for PVD in the study population, and the result is consistent with findings elsewhere [21,37-39].

## Conclusion

The study emphasizes the importance of early detection for PVD and PN among diabetic patients in the UAE, using simple affordable tools and methods. The introduction of such strategy is pivotal in any program aiming to reduce the burden of diabetic foot complications.

### Limitations

The study had few limitations. The study was unable to correlate the clinical findings with electrophysiological and morphologic findings for neuropathy such as nerve conduction studies. Likewise, PVD was not investigated using non-invasive vascular assessments such as the ankle brachial pressure index that may have permitted refining the data. However, despite the limitations mentioned and the limited resources; this first population based survey was able to disclose important information about the diabetic foot problem in the UAE, using simple methods that could be used widely by clinicians to identify foot problems even in remote settings.

### Recommendations

With the presence of high rates of MA in the population (61%) it is imperative to emphasize the importance of regular screening for MA in all diabetic patients, as early screening and treatment are shown to reduce cardiovascular risk, such as neuropathy and nephropathy. Early screening of diabetic patients for hypertension is equally important in order to prevent cardiovascular complications such as PVD. Regular screening for foot complications is recommended to all patients in view of the high rates reported for PN and PVD in the population. Treating physicians should be encouraged to exert more attention and care to foot examination, especially among the elderly and illiterate patients; as ample evidence from the study proves that potential risk factors for foot complications are highly significantly associated with illiteracy.

## Abbreviations

ANOVA-Analysis of variance

BMI-Body Mass Index

DM-Diabetes Mellitus

DNE-Diabetic Neuropathy Examination

DNS-Diabetic Neuropathy Symptoms.

ECG-Electrocardiogram

HDL-High Density Cholesterol

IDF-International Diabetes Federation

IGT-Impaired Glucose Tolerance

LDL-Low Density Cholesterol

MA-Microalbuminuria

PN-Peripheral Neuropathy

PVD-Peripheral Vascular Disease

TC-Total Cholesterol

PHCC-Primary Health Care Centers

WHO-World Health Organization

## Competing interests

The author(s) declare that they have no competing interests.

## Authors' contributions

FA-M conceived the need for the survey in the UAE, participated in its design, and contributed to the interpretation of the results. ME-S participated in the design of the study and the data analysis. FA-M and ME-S collaborated in writing up the manuscript. All authors read and approved the final manuscript.

## Pre-publication history

The pre-publication history for this paper can be accessed here:


